# Advances in lncRNAs from stem cell-derived exosome for the treatment of cardiovascular diseases

**DOI:** 10.3389/fphar.2022.986683

**Published:** 2022-09-06

**Authors:** Jiahui Ma, Pengyu Lei, Haojie Chen, Lei Wang, Yimeng Fang, Xiaoqing Yan, Qinsi Yang, Bo Peng, Libo Jin, Da Sun

**Affiliations:** ^1^ Institute of Life Sciences & Biomedical Collaborative Innovation Center of Zhejiang Province, Wenzhou University, Wenzhou, China; ^2^ Department of Pharmacy, Chinese-American Research Institute for Diabetic Complications, Wenzhou Medical University, Wenzhou, China; ^3^ Wenzhou Institute, University of Chinese Academy of Sciences, Wenzhou, China

**Keywords:** lncRNA, exosome, stem cells, cardiovascular diseases, acellular therapy

## Abstract

Cardiovascular diseases (CVDs) are the leading cause of mortality globally. Benefiting from the advantages of early diagnosis and precision medicine, stem cell-based therapies have emerged as promising treatment options for CVDs. However, autologous or allogeneic stem cell transplantation imposes a potential risk of immunological rejection, infusion toxicity, and oncogenesis. Fortunately, exosome can override these limitations. Increasing evidence has demonstrated that long non-coding RNAs (lncRNAs) in exosome from stem cell paracrine factors play critical roles in stem cell therapy and participate in numerous regulatory processes, including transcriptional silencing, transcriptional activation, chromosome modification, and intranuclear transport. Accordingly, lncRNAs can treat CVDs by directly acting on specific signaling pathways. This mini review systematically summarizes the key regulatory actions of lncRNAs from different stem cells on myocardial aging and apoptosis, ischemia-reperfusion injury, retinopathy, atherosclerosis, and hypertension. In addition, the current challenges and future prospects of lncRNAs treatment for CVDs are discussed.

## Introduction

Cardiovascular diseases (CVDs), which mainly involve the heart and blood vessels (Schmidt, 2019), are the leading cause of morbidity and mortality worldwide (Luo et al., 2018; South et al., 2019). Cases of CVDs increased from 271 million in 1990 to 523 million in 2019, whereas related deaths increased from 12.1 million to 18.6 million (Liu et al., 2021). Currently, surgery and drug are the standard methods for treating CVDs. However, these choices do not enhance the regeneration of damaged myocardial tissue, increasing the chances of recurrence (Stefanini and Holmes, 2013; Rentrop and Feit, 2015). Given that stem cells can differentiate into different mature cell types and possess self-renewal characteristics, stem cell therapy is a potential treatment for CVDs because they induce the regeneration of myocardial cells (Shen et al., 2015; Yamanaka, 2020).

However, the survival rate of transplanted stem cells is very low, decreasing the efficiency of transplantation and the therapeutic efficacy and increases the risk of immune rejection, infusion toxicity, and tumor formation (Liu W. et al., 2020; Zhuang et al., 2020). Recently, numerous studies have confirmed that stem cells mainly exert their effect on CVDs by inducing the secretion of paracrine factors mainly in exosome (Exo) (Elshaer et al., 2018; Terashvili and Bosnjak, 2019; Wu et al., 2020). Although the proportion of long non-coding RNAs (lncRNAs) in Exo is very low (Huang, 2020; Hui et al., 2020; Pham and Boon, 2020), research shows that lncRNAs, especially in stem cell-derived Exo, contribute significantly to treat CVDs by regulating gene expression at the transcriptional level, acting as a molecular sponge that targets miRNA, interfering with chromatin complexes to repress or activate gene expression in an epigenetic fashion and participating the processes of apoptosis, pyrosis, autophagy, myocardial fibrosis, and angiogenesis (Li et al., 2018; Deng et al., 2019; Pan et al., 2019; Yan et al., 2020; Chen et al., 2021a, 2021a; Yuan and Huang, 2021). For example, mesenchymal stem cells (MSCs)-Exo-lncRNA-FENDRR can be taken up by human vascular endothelial cells (HUV-EC-C), where they activate the TEA domain transcription factor 1 (TEAD1) by targeting microRNA (miR)-28 and, thus, inhibits apoptosis, oxidative stress, and inflammatory response of HUV-EC-C, reducing the accumulation of oxidized low-density lipoprotein (ox-LDL), and reduces the formation of atherosclerotic plaques (Zhang N. et al., 2022). In addition, lncRNA-UCA1-rich Exo obtained by hypoxia-stimulated human MSCs secretion inhibit apoptosis *in vivo* and *in vitro via* the lncRNA-UCA1/miR-873-5p↓/X-Linked Inhibitor of Apoptosis Protein (XIAP)↑ axis (Hu et al., 2016; Sun L. et al., 2020). Meanwhile, compared with miRs, lncRNAs have more tissue-specific and developmental stage-specific (Thum and Condorelli, 2015; Zhu et al., 2016).

Adult stem cell (ASC) is more abundant, easier to obtain and does not present ethical dilemmas compared with embryonic stem cells (Barnabé et al., 2009; Shafei et al., 2018; Li et al., 2019; Jain et al., 2020). Therefore, the development of therapeutic approaches to treat CVDs applying ASC-Exo-lncRNA is of utmost importance. This review systematically reviews the research progress and mechanism underlying the function of different ASC-Exo-lncRNAs for CVDs therapy. The challenges and potential clinical application of stem cell-derived Exo-lncRNAs are also discussed.

## ASCs-exo-lncRNAs with diverse organ sources

**FIGURE 1:**
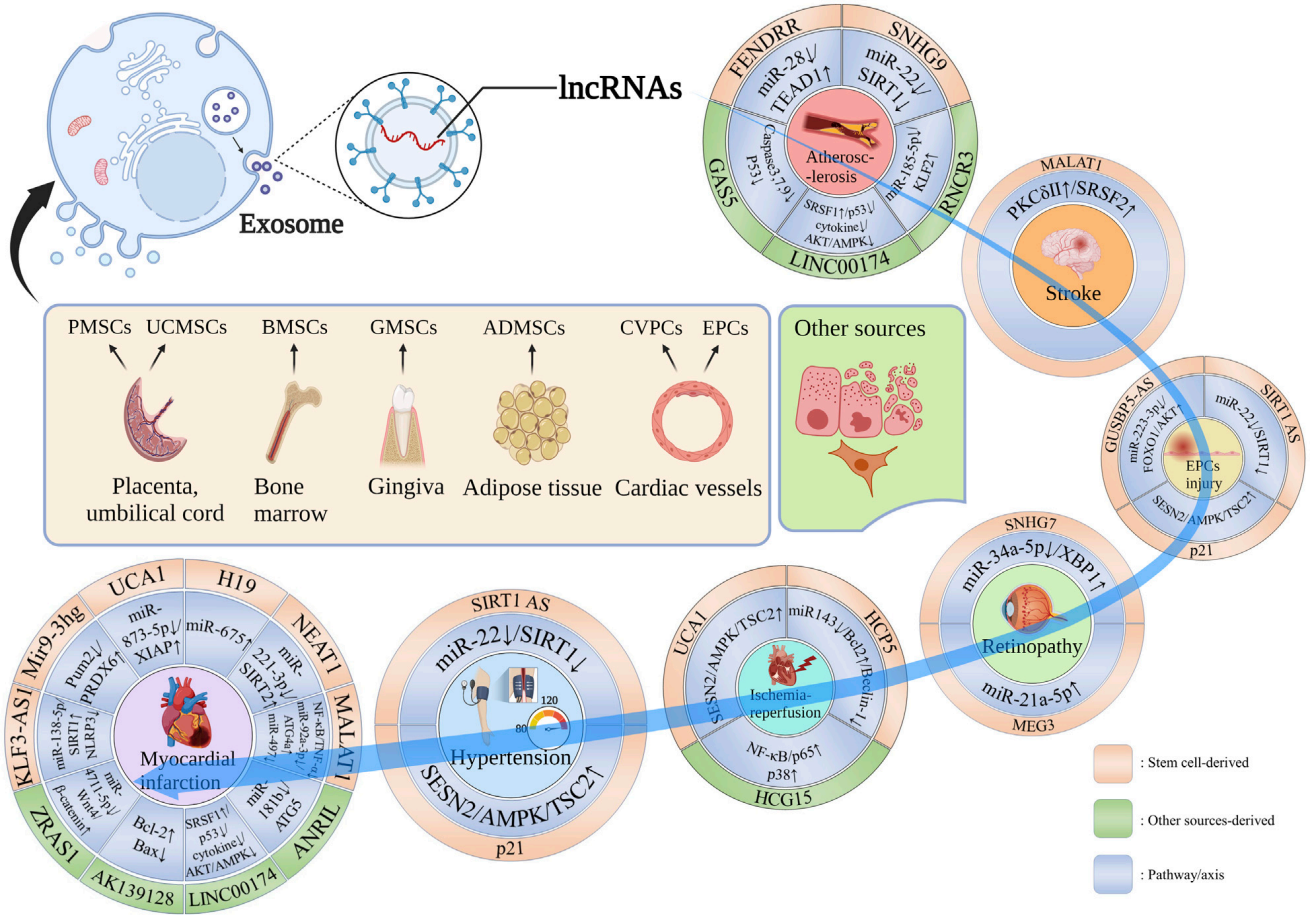
Stem cell-derived Exo-lncRNA with diverse organ sources and other sources in the CVDs occurrence and progression. lncRNA-FENDRR, lncRNA-SNHG9, lncRNA-RNCR3, LINC00174 and lncRNA-GAS5 contribute to AS occurrence and progression; lncRNA-MALAT1 promotes nerve repair after stroke; SIRT1 AS lncRNA, lncRNA-p21 and lncRNA GUSBP5-AS are involved in EPC repair, the former two are also promote vascular damage repair caused by hypertension; lncRNA-SNHG7 and lncRNA-MEG3 are involved in retinopathy; lncRNA-UCA1, lncRNA-HCP5 and lncRNA-HCG15 reduce I/R injury; lncRNA-KLF3-AS1, lncRNA Mir9-3hg, lncRNA-UCA1, lncRNA-H19, lncRNA-NEAT1, lncRNA-MALAT1, lncRNA-ANRIL, LINC00174, lncRNA-AK139128 and lncRNA-ZRAS1 participate in MI process.

As summarized in Figure 1, Exo-lncRNAs derived from bone Marrow (Bone Marrow mesenchymal stem cells, BMSCs), placenta (placental mesenchymal stem cells, PMSCs), adipocyte (adipocyte mesenchymal stem cells, ADMSCs), umbilical cord (umbilical cord mesenchymal stem cells, UCMSCs), gingiva (gingival mesenchymal stem cell, GMSCs) and cardiac vessels (cardiovascular progenitor cells, CVPCs and endothelial progenitor cells, EPCs) have the potential to contribute to CVDs occurrence and progression (Shafei et al., 2018; Gao and Jin, 2020; Jain et al., 2020).

### Cardiovascular protective effect of BMSCs-exo-lncRNAs

The beneficial effect of BMSCs in CVDs has been reported (Afzal et al., 2015). Preventing or reducing cardiomyocyte apoptosis or pyroptosis is necessary to ensure normal cardiac contractile function. Mao et al. (Mao et al., 2019) found that BMSCs- Exo overexpressing lncRNA-KLF3-AS1 in hypoxic cardiomyocytes and rats could improve the morphology of cardiomyocytes and inhibit the inflammatory response induced by pyroptosis. Meanwhile as a competitive endogenous RNA of sponge miR-138-5p, lncRNA-KLF3-AS1 mediates the expression of sirtuin 1 (SIRT1) and inhibits the activation of NOD-like receptor family pyrin domain containing 3 (NLRP3) inflammatory bodies and, thus, regulates the pyroptosis of cardiomyocytes and the progression of miocardial infarction (MI).

An increase of miR-497 during ischemia-reperfusion (I/R) injury may cause cardiomyocyte apoptosis. Li et al. (Li K.-S. et al., 2021) confirmed that Introducing Exo-lncRNA-HCP5 in hBMSCs into cardiomyocytes can protect cardiomyocytes from injury *via* the miR-497↓/insulin like growth factor-1 (IGF-1)/phosphatidylinositide 3-kinases (PI3K)/protein kinase B (AKT)↓ signal pathway. Meanwhile, Zhang et al. (Zhang J.-K. et al., 2022) treated HL-1 mouse cardiomyocytes and myocardial tissue of hypoxia reperfusion (H/R) myocardial cells ferroptosis mouse model with BMSCs-Exo-lncRNA-Mir9-3hg. The results showed that BMSCs-Exo-lncRNA-Mir9-3hg inhibits the upregulation of pumilio RNA binding family member 2 (Pum2), promotes glutathione content, peroxiredoxin 6 (PRDX6), the proliferation of HL-1 mouse cardiomyocytes, and inhibits the iron concentration, production of reactive oxygen species, and acyl CoA synthetase long chain family member 4 expression in HL-1 cells treated with H/R injury. In addition, high glucos initiated proliferation and migration of retinal endothelial cells, which is a critical step of diabetic retinopathy (DR) development. Cao et al. (Cao et al., 2021) found that human BMSCs-Exo transduces lncRNA-SNHG7 into human retinal microvascular endothelial cells (HRMECs) and inhibits the endothelial mesenchymal transformation and tubule formation of HRMECs *via* the miR-34a-5p↓/X-box binding protein 1 (XBP1)↑ (a transcription factor associated with endoplasmic reticulum stress regulation) axis. Accordingly, this axis is a feasible target for treating pathological fibrosis in DR.

Apart from the above, MSCs can enhance its protective effect on myocardial function after MI following appropriate drug treatment, such as atorvastatin (ATV) and migration inhibitory factor (MIF) (Li et al., 2015, 4; Liu X. et al., 2020). Huang et al. (Huang P. et al., 2020) treated BMSCs with ATV to obtain Exo overexpress lncRNA-H19 (MSC^ATV^-Exo). The lncRNA regulated the activation of vascular endothelial growth factor and intercellular adhesion molecule-1 in endothelial cells and cardiomyocytes by targeting miR-675↑. lncRNA-H19 suppresses inflammation, promotes healing of infarct damage, reduces cardiomyocyte apoptosis, promotes angiogenesis, and elongates the endothelial cell survival in rat acute MI model and, thus, improves cardiac function. Concurrently, BMSCs-Exo overexpressed the lncRNA-NEAT1 by MIF treatment which had anti-aging effects on Dox-induced cardiomyopathy (DIC). On the contrary, silencing lncRNA-NEAT1 inhibited the effect of Exo on DIC^MIF^, and this is because Exo^MIF^ attenuates cardiomyocyte senescence induced by Dox *via* the Exo/lncRNA-NEAT1↑/miR-221-3p↓/sirtuin 2 (SIRT2)↑ pathway (Zhuang et al., 2020). These findings provide an important reference on how to improve the role of lncRNAs.

### ADMSCs-exo-lncRNAs reverse cardiomyocyte senescence and apoptosis and promote nerve repair after stroke

ADMSCs are easily obtained from adipose tissue (Shafei et al., 2018), and the Exo in supernatant enhances angiogenesis (Almeria et al., 2019). Xia et al. (Xia et al., 2020) observed that hypoxia-induced ADMSCs-Exo-lncRNA-MALAT1 improves mitochondrial metabolism by regulating the miR-92a-3p↓/autophagy related genes 4a (ATG4a)↑ axis. Also, miR-92A-3p plays a cardioprotective role in DIC. ADMSCs-Exo-lncRNA-SNHG9 modulates inflammation by inhibiting endothelial cell apoptosis *via* the nuclear factor kappa-B (NF-κB)↓/TNF receptor type 1-associated death domain protein (TRADD)↓ pathway (Song et al., 2020). ADMSCs-Exo-lncRNA-SNHG9 is a potential therapeutic target for CVDs related to lipid metabolism and, thus, for AS treatment. In addition, MIF treatment induces the overexpression of lncRNA-NEAT1 in ADMSC-Exo which could prevent cardiomyocyte apoptosis induced by H_2_O_2_ *via* the lncRNA-NEAT1↑/miR-142-3p↓/Forkhead box O1 (FOXO1)↑ pathway. Also, lncRNA-NEAT1 can regulate oxidative stress and protect against neural injury (Chen H. et al., 2020), providing a new signaling pathway target for improving MI therapy.

On the other hand, given their unique self-renewal and differentiation abilities, stem cells have been designed to treat stroke (Chen H.-X. et al., 2020; Singh et al., 2020). Improving neural repair and recovery in the postacute phase of stroke may reduce the overall long-term burden of stroke (Murie-Fernández and Marzo, 2020). El Bassit et al. (El Bassit et al., 2016) revealed that human ADMSCs-Exo increases the expression of protein kinase CδⅡ (PKCδⅡ) on immortalized mouse hippocampal cell line (HT22) after injury and promotes the survival and proliferation of neurons. lncRNA-MALAT1 promotes alternative splicing of PKCδII, which increases the survival of neurons by inducing the recruitment of serine-arginine-rich splicing factor 2 (SRSF2). Meanwhile, insulin could further enhance the effect with lncRNA-MALAT1 application. Stroke treatment may be improved as a result of this research.

### UCMSCs-exo-lncRNAs ameliorate the H/R and myocardial aging injury

UCMSCs have been exploited for treating CVDs and depend on paracrine effect (Chen et al., 2021b; Chang et al., 2021). For instance, human UCMSCs (hUCMSCs)-Exo prevents the apoptosis of cardiomyocytes and promotes tubular formation and migration of umbilical vein endothelial cells (Zhao et al., 2015). At the same time, hUCMSCs-Exo-lncRNA-UCA1 enhances the proliferation, invasion, migration of cardiac microvascular endothelial cells (CMECs) and inhibits the apoptosis and autophagy of CMECs caused by H/R *via* the miR143↓/B-cell lymphoma-2 (Bcl-2)↑/Beclin-1↓ axis (Diao and Zhang, 2021). Furthermore, Zhu et al. (Zhu et al., 2019) reported that among the lncRNAs that may possess anti-aging properties, only lncRNA-MALAT1 is highly expressed in Exo. HUCMSCs-Exo-lncRNA-MALAT1 can prevent cardiac dysfunction arising from aging through the NF-κB/tumor necrosis factor (TNF-α)↓ pathway. Meanwhile, lncRNA-MALAT1 silencing significantly reduces the anti-aging effect of Exo.

### Exo-lncRNAs from PMSCs affect acute MI

Death of many cardiomyocytes causes strong inflammation after MI, and studies have shown that intestinal microflora participates in the occurrence of this kind of inflammation (Wang et al., 2018; Zununi Vahed et al., 2018). (Yang et al., 2022) pointed out that PMSCs-Exo shows angiogenesis and anti-inflammatory potential in the cell therapy of MI and regulates the intestinal microflora. Gene ontology enrichment analysis of the PMSCs-Exo-lncRNA target gene revealed that lncRNA performs numerous functions at the transcriptional level, suggesting that PMSCs-Exo-lncRNA is a potential target for MI therapy.

### Exo-lncRNAs from GMSCs protect nerves in retina I/R

GMSCs which not only show the potential for self-renewal and multi-differentiation but also have immunomodulatory, anti-inflammatory, and effective tissue regeneration properties can easily be obtained from gum tissues (Liu et al., 2015; Al-Qadhi et al., 2021). MiR-21-5p overexpressed in TNF-α-stimulated GMSCs-Exo which reduces inflammation and death of mouse primary retinal ganglion cells and microglia simultaneously. Vitreous injection of GMSCs-Exo alleviated retinal I/R injury in mice induced by high intraocular pressure *via* the Exo-lncRNA-MEG3↑/miR-21-5p↑ axis (Yu et al., 2022). This is a potential target for glaucoma treatment and other retinal neuroinflammatory diseases.

### CVPCs-exo-lncRNAs and EPCs as theranostic strategies for CVDs

CVPCs-Exo injected into the myocardium significantly improved the cardiac function of mice with acute MI. Moreover, overexpression of lncRNA-MALAT1 in hypoxic preconditioning Exo increases the viability of neonatal rat cardiomyocytes (NRCMs) damaged by oxygen and glycogen deprivation, and lncRNA-MALAT1 gene knockout inhibits tubular formation of human umbilical endothelial cells (HUVECs) promoted by CVPCs-Exo. In addition, lncRNA-MALAT1 improved the survival of NRCMs and HUVECs’ formation by targeting miR-497 (Wu et al., 2020). Therefore, hypoxic preconditioning CVPCs-Exo could be used for treating MI with high lncRNA expression and promise option for cardiac repair. However, more basic research is required to understand their mechanism of action (Zhang et al., 2016).

On the other hand, hypertension induces autophagy due to the pressure on the vascular wall to maintain intracellular stability, and the reduction in autophagy causes angiotensin Ⅱ (AngⅡ)-induced senescence and damage to EPCs (Bianconi et al., 2018). EPCs-Exo-lncRNA-p21 can activate the sestrin 2 (SESN2)/AMP-activated protein kinase (AMPK)/tuberous sclerosis 2 (TSC2) pathway and enhance autophagy to prevent AngII-induced EPCs injury by promoting the transcriptional activity of p53 (Li C. et al., 2021). Meanwhile, stimulation of EPCs using niacinamide phosphoribosyltransferase upregulates the expression of SIRT1 and SIRT1 antisense long non-coding RNA (SIRT1 AS lncRNA). This overexpression of SIRT1 AS lncRNA in EPCs upregulates that of SIRT1, and inhibiting miR-22 abrogated the aging of EPCs and promoted the proliferation and migration of EPCs (Ming et al., 2016). In addition, regarding the clinically upregulated expression of lncRNA GUSBP5-AS (Enst00000511042) in EPCs of deep venous thrombosis patients, Sun et al. (Sun L.-L. et al., 2020, 1) revealed that lncRNA GUSBP5-AS regulates the expression of fibroblast growth factor 2 and matrix metalloproteinase 2/9 through the miR-223-3p↓/FOXO1/AKT↑ pathway and subsequently regulates angiogenesis, as well as proliferation and homing capacity of EPCs. Therefore, EPCs-Exo-lncRNA is a potential therapeutic target for vascular endothelial repair.

## Exo-lncRNA from other sources as potential target lncRNAs for CVDs

Stem cell-derived Exo-lncRNA has shown excellent potential in treating CVDs. In fact, several Exo-lncRNAs which are contained in something else can also treat CVDs. For instance, Exo-lncRNA-RNCR3 in HUVECs which could regulates the dysfunction of endothelial cells and vascular smooth muscle cells (VSMCs) by targeting the miR-185-5p↓/kruppel-like factor (KLF)2↑ axis (Shan et al., 2016), highly expressed Exo-LINC01005 of ox-LDL-treated HUVECs which could regulate the miR-128-3p↓/KLF4↑ axis to promote the proliferation and migration of VSMCs (Zhang Z. et al., 2020), and the lncRNA-GAS5 derived from human acute monocytic leukemic cell line (THP-1) which could reduce the apoptosis of HUVECs *via* up-regulated the expressions of P53, Caspase 3, Caspase 7 and Caspase 9 (Chen et al., 2017) all participate in the occurrence and development of AS. Otherwise, Exo-lncRNA-ZRAS1 from human cardiomyocytes which could promote cardiac fibrosis *via* the miR-4711-5p↓/Wnt4/β-catenin↑ signaling pathway (Wang et al., 2021), hypoxia-induced cardiac myocytes (CMs) overexpressing Exo-lncRNA-AK139128 which could inhibit cardiac fibroblasts (CFs) proliferation and migration, elevates CFs apoptosis *via* increased level of Bcl-2 while decreased expression of Bax (Wang and Zhang, 2020), Exo-LINC00174 with high expression in endothelial cells which could inhibit apoptosis, vacuole, and autophagy of CMs *via* the SRSF1↑/p53↓/myocardin↓/AKT/AMPK↓ signaling pathway (Su et al., 2021) and Exo-lncRNA-ANRIL expression increased in CMECs treated with indoxyl sulfate which could be absorbed by CMs to increase autophagy, whereas recombinant autophagy-related gene 5 (ATG5) expression can be reduced in CMs by silencing ANRIL or upregulating miR-181b, thereby reversing the autophagy of CMs in uremic mice (Xu et al., 2021, 5) provide reference for the intervention of MI in order to obtain good therapeutic effect. Moreover, under hypoxia conditions, AC16 CMs express a high level of lncRNA-HCG15, which stimulates apoptosis, releases inflammatory factors, inhibits cell proliferation, and aggravates I/R injury in C57BL/6 J mice *via* the NF-κB/p65↑ and p38↑ pathways (Lin et al., 2021). Together, studies such as these could provide a new direction for early diagnosis and targeted treatment of CVDs.

## Challenges in the applications of stem cell-derived Exo-lncRNA

**FIGURE 2:**
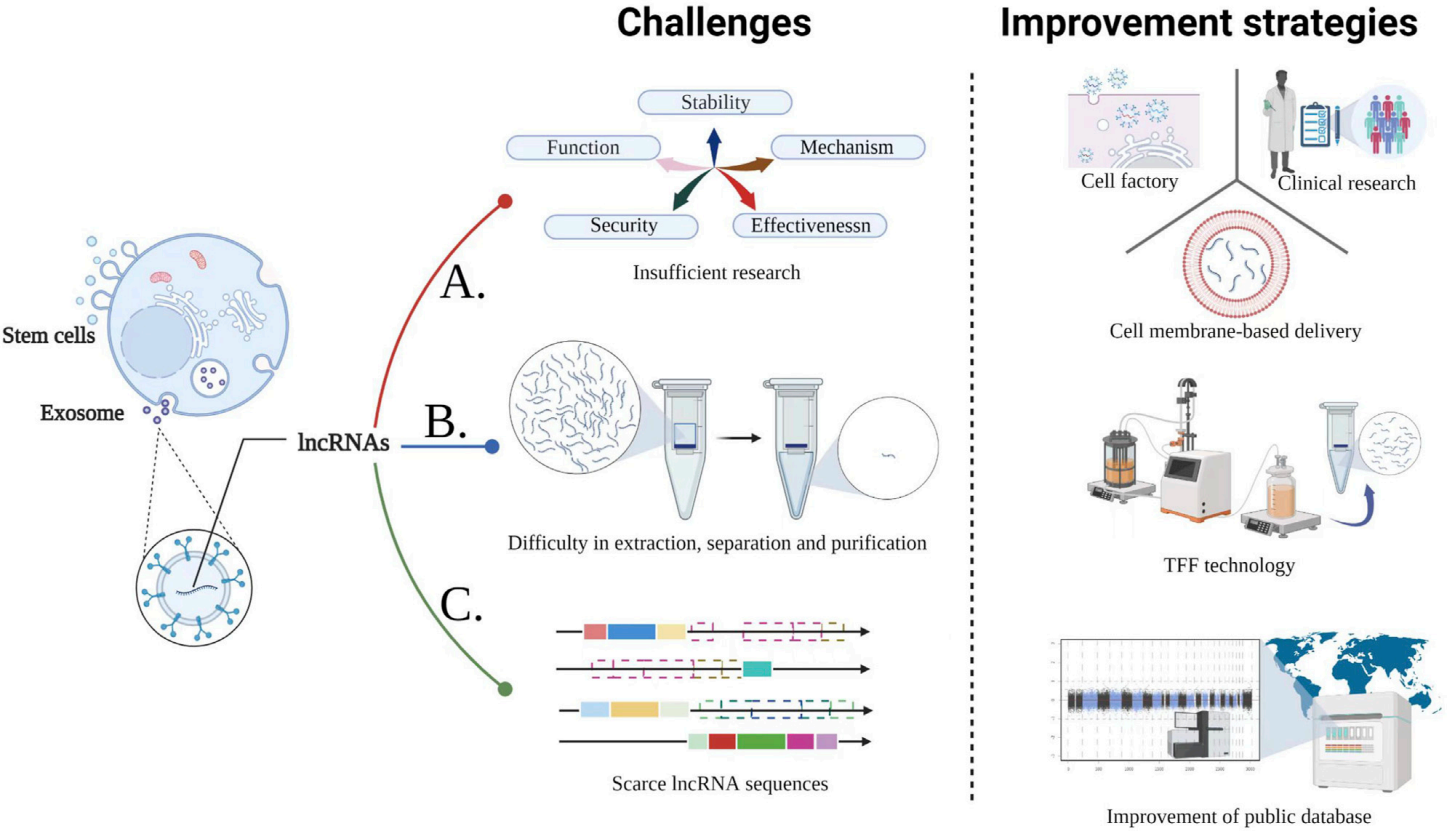
Challenges in the CVDs treatment with stem cell-derived Exo-lncRNA and the corresponding improvement strategies. A. The following three methods provide guidance for overcoming the deficiencies in studies related to the function, mechanism, stability, safety, and effectiveness of stem cell-derived Exo-lncRNA; B. TFF technology is efficient for large-scale production of lncRNA; C. Eatablishing and improving public databases will assist in solving the limitations of scarce lncRNA sequences, reducing confusion and improving convenience.

Notably, there are several challenges in the applications of stem cell-derived Exo-lncRNA (Figure 2). The serum levels of lncRNA-LUNAR1 in patients with chronic total coronary occlusion are closely related to the development of coronary blood supply and collateral (Lu et al., 2020). Nevertheless, the causal relationship and whether it can be used as a treatment remains unclear. In addition, Further studies are needed to evaluate the clinical efficacy and safety of lncRNAs in CVDs, as well as their potential downstream targets (including different cell types and pathways), mechanisms of action and potential risks (such as off-target effects) (Dong et al., 2019; Braga et al., 2020; Chen et al., 2021a). At present, the standard procedure for purification of Exo or lncRNAs needs to be optimized if these molecules are to be applied in clinical treatment (Ma et al., 2019; Zhang W. et al., 2020). Meanwhile, the cell factory approaches for Exo simulations with specific miRs and lncRNAs in the self environment may be an alternative strategy to overcome the limitations of stability and potential immunogenicity (Rotini et al., 2018). In recent years of membrane-based delivery systems (such as erythrocyte, the advantage membranes) over nanoparticle drugs have been extensively demonstrated (Sun et al., 2019; Zhu et al., 2022). Consequently, the foundation is laid for developing lncRNA drug delivery systems for CVDs that are more stable and targeted. In addition, The separation rate of UCMSCs-Exo using tangential flow filtration (TFF) is 92.5 times more efficient than using the ultracentrifuge (UC)-based conventional method (Kim et al., 2021). TFF can therefore be used for mass purification of Exo-lncRNAs. Remarkably, lncRNA sequences in the published studies are scarce, and not all studies provide chromosome mapping. To minimize confusion and to facilitate the use and replication of data, more details should be provided (i.e. publicly public databases for lncRNA sequence data is needed) (Uchida and Dimmeler, 2015). Significantly, research has revealed dysregulated expression of 768 lncRNAs in the plasma of patients with MI (Lu and Thum, 2019). RNA sequencing data of epicardial adipose tissue collected from 6 atrial fibrillation, and 6 sinus rhythm showed that eight lncRNAs including LINC00694 are closely related to TNF-α signaling pathways demonstrating their broad application potential. Aside from biomedical functions, Exo-lncRNA delivery systems have a greater proliferation of targets than antibodies or small molecules, due to their proximity and tissue specificity, resulting in fewer off-targets of lncRNAs/miRs (Huang Y. et al., 2020, Huang et al., 2020 C.-K.; Zhao et al., 2020). Even though lncRNA is infant in clinical applications, it appears that it has substantial potential in this aspect, considering the clinical cases of miRs which utilize inhibitors and are delivered by endothelial microparticles delivery (Barwari et al., 2016; Nakaoka et al., 2018; Täubel et al., 2021). At present, although the specific differences of Exo-lncRNAs in stem cells from different sources are not clear, however, given ADMSC has extremely abundant yield, inhibits the growth of cancer *in vivo* and is less affected by aging, doubling quantity, and other negative factors, and ADMSC-Exo with exogenous factors has great therapeutic effects (Maguire, 2019; Shin et al., 2021). So going forward, ADMSCs-Exo-lncRNA will stand out in the clinical translation of CVDs treatment. In conclusion, these results are sufficient evidence that lncRNA can be applieed for a broad range of clinical diagnoses and applications.

## Conclusion

Exo-lncRNA-based therapeutic strategies is novel but still in infancy. Nevertheless, the recent developments of Exo-lncRNA in CVDs have demonstrated their superior properties for early diagnosis and targeted therapy, thereby promoting the potential transition from bench to bedside. Presently, stem cell-derived Exo-lncRNAs are gradually reducing their application limitations as technology advances, bioinformatics improves, and drug delivery strategies are continuously improved. Hence, these advantages ushered in a new dawn for the clinical application of stem cell-derived Exo-lncRNA. Determining the therapeutic efficacy and safety of Exo-lncRNA can accelerate their use for treating CVDs.

## Glossary

ADMSCs: Adipocyte mesenchymal stem cells
AKT: Protein kinase B
AngⅡ: Angiotensin Ⅱ
AMPK: AMP-activated protein kinase
ATG5: autophagy-related gene 5
ASCs: Adult stem cells
ATG4a: Autophagy related genes 4a
ATV: Atorvastatin
Bcl-2: B-cell lymphoma-2
BMSCs: Bone marrow mesenchymal stem cells
CMs: Cardiac myocytes
CFs: Cardiac fibroblasts
CMECs: Cardiac microvascular endothelial cells
CVDs: Cardiovascular diseases
CVPCs: Cardiovascular progenitor cells
DR: Diabetic retinopathy
DIC: Dox-induced cardiomyopathy
EPCs: Endothelial progenitor cells
Exo: Exosome
EAT: Epicardial adipose tissue
FOXO1: Forkhead box O1
GMSCs: Gingival mesenchymal stem cells
HUV-EC-C: Human vascular endothelial cells
H/R: Hypoxia reperfusion
HRMECs: Human retinal microvascular endothelial cells
hUCMSCs: Human UCMSCs
KLF: Kruppel-like factor
NRCMs: Neonatal rat cardiomyocytes
HUVECs: Human umbilical endothelial cells
I/R: Ischemia-reperfusion
IGF-1: Insulin like growth factor-1
lncRNAs: Long non-coding RNAs
MSCs: Mesenchymal stem cells
MI: Myocardial infarction
MIF: Migration inhibitory factor
miR: MicroRNA
NF-κB: Nuclear factor kappa-B
PMSCs: Placental mesenchymal stem cells
Pum2: Pumilio RNA binding family member 2
PI3K: phosphatidylinositide 3-kinases
PRDX6: Peroxiredoxin 6
PKCδⅡ: Protein kinase CδⅡ
ox-LDL: Oxidized low-density lipoprotein
SESN2: Sestrin 2
SIRT1: Sirtuin 1
SIRT2: Sirtuin 2
SRSF2: Serine-arginine-rich splicing factor 2
SIRT1 AS lncRNA: SIRT1 antisense long non-coding RNA
TEAD1: TEA domain transcription factor 1
TFF: Tangential flow filtration
TNF-α: Tumor necrosis factor-α
TSC2: Tuberous sclerosis 2
UC: Ultracentrifuge
UCMSCs: Umbilical cord mesenchymal stem cells
VSMCs: Vascular smooth muscle cells
XIAP: X-Linked Inhibitor of Apoptosis Protein
XBP1: X-box binding protein 1
